# Is Altruistic Behavior Associated with Major Depression Onset?

**DOI:** 10.1371/journal.pone.0004557

**Published:** 2009-02-23

**Authors:** Takeo Fujiwara

**Affiliations:** Section of Behavioral Science, Department of Health Promotion and Research, National Institute of Public Health, Wako-shi, Saitama, Japan; Aga Khan University Karachi, Pakistan

## Abstract

**Background:**

Previous cross-sectional study showed altruistic behaviors were harmful on major depression (MD). It is needed to investigate the impact of altruistic behaviors by its contents on the development of MD prospectively.

**Methodology/Principal Findings:**

The National Survey of Midlife Development in the United States (MIDUS) in 1995–1996 and the MIDUS Psychological Experience Follow-Up study in 1998 were analyzed (weighted N = 563). Financial support of 10 or more dollars per month had a significant impact on the development of MD in comparison to no financial support (OR: 2.64, 95% CI: 1.05–6.62). Unpaid assistance and providing emotional support were not significantly associated with the development of MD in later life.

**Conclusions/Significances:**

Those who provide financial contribution to individuals other than family members can be at risk of developing MD.

## Introduction

The estimated prevalence of depression was 9.6% in the United States. This prevalence was the highest among 14 countries (6 less developed and 8 developed) in the World Health Organization World Mental Health Initiative [1]. The burden of depression in the United States is estimated to be $43.7 billion [2]. By examining risk and protective factors, it would be possible to determine what types of behaviors are related to the development of depression. One type of behavior to be examined are altruistic behaviors (AB), which is defined as “behavior intended to benefit another, even when this risks possible sacrifice to the welfare of the actor” [3]. According to positive psychology, AB can regulate people's perception of those internal and external realities they are powerless to change, and empower them to effect meaningful change [4]. Literature on caregiving suggests that the caregiver, who is considered as an actor of AB, might receive rewards due to the perception of doing something good for others [5]. On the other hand, the actor of AB might feel stressed when the sacrifice of the actor is too large.

Previous studies reporting a link between AB and mental health showed mixed directions. Schwartz *et al.* (2003) found that those who do something for others showed better mental health. Fujiwara (2007) reported that altruistic behavior had a tendency to protect generalized anxiety disorder, but was harmful for major depression (MD). However, these studies used cross-sectional design. Brown et al reported more beneficial effects of providing social support than receiving it by prospective study [6]; however, as they measured the outcome as mortality, the impact on mental health remains uncertain. In addition, the impacts of AB on mental health depending on the types of AB were not well-reported. In the study of AB towards children and grandchildren by parents and grandparents, fathers and grandfathers who provide moderate amounts of informal assistance and financial support to children and grandchildren were protected to develop MD in 2–3 years, although not among mothers and grandmothers [7]. Thus, there is a need to investigate the impact of AB towards others (i.e., non-family member) on the development of MD of AB providers. The purpose of this study is to investigate the impact of AB toward others on the development of MD prospectively.

## Methods

### Participants

For baseline data, the author reanalyzed the publicly-available National Survey of Midlife Development in the United States (MIDUS) data originally collected in 1995–1996 as a collaborative, interdisciplinary investigation of the patterns, predictors, and consequences of midlife development in the areas of physical health, psychological well-being, and social functioning [8], although the author was not involved in the original study. Respondents were selected from a nationally representative, random digit dial sample of non-institutionalized, English-speaking adults, between 25 and 74 years of age, selected from working telephone banks in the coterminous United States. Detailed information regarding the MIDUS study has been published previously [9]. The sample for the current analysis includes original MIDUS respondents who completed both the telephone survey (response rate 70 percent), which included demographic characteristics and Composite International Diagnostic Interview Short Form (CIDI-SF) based diagnoses of MD, as well as the postal questionnaire (response rate 87 percent), which captured information related to AB. The combined response rate for both the telephone survey and the postal questionnaire was 60.8%. Follow-up data was obtained using the Psychological Experiences Follow-Up Study implemented in 1998 [10]. The primary objective of the follow-up study was to explore how adults perceive psychological change in their lives. This study was a random telephone follow-up of 724 respondents of the original MIDUS random-digit-dial sample (82% response rate among 883 participants selected from the original MIDUS survey who were re-contacted). After explaining the study to the informant, a household listing was generated of people in the age range of 25 to 74, and a random respondent was selected. Men and older people were oversampled. To see the impact of AB on the development of MD in later life, respondents who had MD at baseline were excluded from analysis sample, resulting in N = 639. In addition, to adjust for possible selection bias and differential non-responses, sampling weights were applied for all analysis yielding weighted N = 563.

### Assessment of Major Depression

MIDUS researchers assessed MD in both the original and follow-up study using CIDI-SF [11]. The diagnosis of MD was based on definitions and criteria specified in the DSM-III-R, comprised of 19 items [12]. A diagnosis of MD requires a period of at least two weeks of either depressed mood or anhedonia most of the day, nearly every day, and a series of at least four other associated symptoms typically found to accompany depression, including problems with eating, sleeping, energy, concentration, feelings of low self-worth, and suicidal thoughts or actions. MD was assessed by telephone interview in both the original and follow up surveys. In the original MIDUS survey, the time frame for inquiring about MD symptoms was the previous 12 months, while in the follow-up MIDUS survey, MD items inquired about the past 5 years to capture all depressive episodes during follow-up. The test-retest reliability and clinical validity of CIDI-SF diagnoses have previously been examined and found to be high [13]. Psychometric properties are also acceptable: sensitivity was 0.73 and specificity was 0.82 in comparison with semi-structured clinical diagnostic interview [14]. Moreover, the MD scales employed in the present study were used in a previous publication, where it was based upon the responses of the MIDUS dataset [7], [15], [16].

### Measurement of Altruistic Behaviors

AB was measured by mail-in questionnaire in three dimensions: unpaid assistance, emotional support, and financial support. Unpaid assistance was assessed by asking the following question: “On average, about how many hours per month do you spend providing unpaid assistance, such as help around the house, transportation, or childcare, to anyone else other than family members or close friends?” Emotional support was assessed by the following question: “On average, about how many hours per month do you spend giving informal emotional support, such as comforting, listening to problems, or giving advice to anyone else other than family members or close friends?” For the question of unpaid assistance and emotional support, respondents answered by the number of hours. Financial support was measured by the following question: “On average, about how many dollars per month do you or your family living with you contribute to any individuals other than family members or close friends, including people on the street asking for money? If you contribute food, clothing or other goods, include their dollar value.” Respondents answered by the amount of dollars. AB measurements were further categorized into three, non-AB, low AB, and high AB. First, those who did not engage in AB were categorized as non-AB. Second, those who provided AB were divided into two groups, low AB and high AB. Cut-off of low and high AB was decided based on their distributions. Consequently, unpaid assistance was categorized as 0, 1–2, and 3 or more hours/months; weighted distribution was 64.7%, 17.1%, and 18.2%, respectively. Emotional support was categorized as 0, 1–4 and 5 or more hours/month; weighted distribution was 43.2%, 27.6%, and 29.1%, respectively. Financial support was divided into 0, 1–9 dollars and 10 and more dollars per month; weighted distribution was 82.2%, 8.3%, 9.6%, respectively.

### Measurement of potential confounders

Based on previous findings [7], [15], [17], age, sex, race, education, working status, and marital status were considered as potential confounders of the association between AB and MD. Age was categorized as 25–34, 35–44, 45–54, 55–64, and 65–74 years old for further analysis. Race was categorized as Whites, Blacks, and Others (Native American, Aleutian Islander/Eskimo, Asian, Pacific Islanders, multiracial, or other). Education was categorized into 4 groups: less than high school, graduated high school, some college, and graduate college or more. Working status was categorized as full-time working, retired, homemaker, and unemployed. Marital status was categorized as married, separated, divorced, widowed, and unmarried.

### Statistical Analysis

First, association between AB and demographic variables were examined by ordered logistic regression. Second, unadjusted and adjusted odds ratio of AB at baseline on MD in follow-up were calculated. In adjusted model, all AB measurements and demographic variables were included as explanatory variables. It is assumed that the association has an independent and significant effect if *P*<0.05 (two-sided). All analyses were undertaken using Stata SE, version 9 (Stata Corp., College Station, Texas).

## Results

Demographic characteristics and prevalence of MD in 1998 of the study sample were shown in Table 1. Regarding demographic characteristics, 55 percent of the sample was women, 86 percent white, 90 percent high school graduates or more, 63 percent full-time workers, and 75 percent married. Among those who did not have MD at baseline, in 1995–1996, 11.7% of them developed MD in 1998 (95% confidence interval [CI]: 8.59%–14.8%).

**Table 1:** Characteristics of sample (weighted N = 563).

		Weighted %
Gender	Female	55.1
	Male	44.9
Age (years old)	25–34	17.4
	35–44	28.5
	45–54	17.8
	55–64	20.5
	65–74	15.8
Race	White	85.9
	Black	9.8
	Others	4.2
Education	<High school	10.3
	High school	41.3
	Some college	24.8
	≥College	23.7
Working Status	Full-time working	62.9
	Retired	16.9
	Homemaker	11.9
	Unemployment	8.2
Marital Status	Married	74.6
	Separated	1.4
	Divorced	9.2
	Widowed	6.2
	Never married	8.7
Major Depression in 1998	Yes	11.7

Table 2 shows the weighted odds ratio of demographic characteristics on AB. Higher education level was associated with providing unpaid assistance and emotional support. High school graduates, some college, college or more graduates were significantly providing unpaid assistance in comparison with those who did not graduate high school. Similarly, some college and college or more graduates showed a higher likelihood of providing emotional support, compared with those who did not graduate high school. However, financial support was not associated with education level. Older age groups were less likely to provide unpaid assistance and emotional support; while no such association was found for financial support. Race as other (Native American, Aleutian Islander/Eskimo, Asian, Pacific Islander, multiracial, or other) than white or black were 4.3 times more likely to provide financial support compared with whites (95% CI: 1.86–9.86). Separated marital status were associated with higher provision of financial support (odds ratio [OR]: 3.58, 95% CI: 1.28–10.0). Gender and working status were not associated with any type of AB.

**Table 2:** Weighted odds ratio of demographic characteristics on altruistic behaviors by ordered logistic regression.

		Unpaid assistance	Emotional support	Financial support
Gender	Female	0.82 (0.55–1.21)	1.36 (0.94–1.97)	0.78 (0.48–1.27)
	Male	Reference	reference	reference
Age (years old)	25–34	Reference	reference	reference
	35–44	0.76 (0.41–1.42)	0.81 (0.45–1.48)	1.20 (0.53–2.73)
	45–54	0.63 (0.33–1.18)	0.45 (0.25–0.81)**	1.42 (0.61–3.32)
	55–64	0.53 (0.28–1.00)	0.51 (0.28–0.93)*	1.55 (0.66–3.63)
	65–74	0.78 (0.34–1.78)	0.53 (0.24–1.16)	0.36 (0.13–1.02)
Race	White	Reference	reference	reference
	Black	0.52 (0.21–1.27)	0.65 (0.25–1.69)	1.84 (0.73–4.66)
	Others	0.84 (0.34–2.09)	0.79 (0.35–1.80)	4.28 (1.86–9.86)**
Education	<High school	Reference	reference	reference
	High school	3.10 (1.21–7.96)*	1.99 (0.81–4.89)	0.79 (0.28–2.29)
	Some college	4.62 (1.82–11.7)**	3.09 (1.27–7.56)*	1.45 (0.53–3.99)
	≥College	3.79 (1.50–9.60)*	2.45 (1.04–5.80)*	2.35 (0.87–6.35)
Working Status	Full-time working	Reference	reference	reference
	Retired	1.31 (0.74–2.31)	0.82 (0.47–1.43)	0.67 (0.34–1.33)
	Homemaker	1.54 (0.82–2.90)	1.90 (0.95–3.82)	0.82 (0.32–2.07)
	Unemployment	1.34 (0.69–2.59)	1.22 (0.64–2.34)	1.03 (0.46–2.29)
Marital Status	Married	Reference	reference	reference
	Separated	0.80 (0.25–2.55)	1.30 (0.53–3.19)	3.58 (1.28–10.0)*
	Divorced	0.84 (0.43–1.65)	0.74 (0.45–1.23)	0.45 (0.15–1.33)
	Widowed	1.39 (0.58–3.32)	0.97 (0.44–2.13)	0.35 (0.11–1.13)
	Never married	0.88 (0.39–2.02)	0.99 (0.39–2.54)	1.02 (0.45–2.31)

* p<0.05.

** p<0.01.

Table 3 describes weighted unadjusted and adjusted odds ratio of AB on MD in 2–3 years later. Unpaid assistance, emotional support, and financial support were not associated with MD in unadjusted model. However, in adjusted model, financial support were significantly associated with MD onset: those who provide 10 or more dollars/month to anyone else other than family member or close friend were 2.6 times significantly more likely to develop MD in 2–3 years (OR: 2.64. 95% CI: 1.05–6.62). Unpaid assistance was not associated with MD onset in adjusted model: although point estimate showed a protective direction. Emotional support was not associated with MD onset either, although point estimate showed harmful direction.

**Table 3:** Weighted unadjusted and adjusted odds ratio of altruistic behaviors on major depression in 2–3 years later.

Variables		unadjusted analysis	adjusted analysis
Altruistic behaviors			
Unpaid assistant (hour/month)	0	reference	reference
	1–2	1.08 (0.51–2.31)	0.91 (0.39–2.15)
	3+	0.54 (0.20–1.46)	0.38 (0.12–1.16)
Emotional support (hour/month)	0	reference	reference
	1–4	1.06 (0.51–2.22)	1.11 (0.47–2.61)
	5+	1.75 (0.84–3.65)	2.04 (0.83–5.03)
Financial support (dollar/month)	0	reference	reference
	1–9	1.49 (0.52–4.26)	1.68 (0.56–5.09)
	10+	2.16 (0.97–4.80)	2.64 (1.05–6.62)*
Demographics			
Gender	Female/Male		2.62 (1.31–5.23)**
Age^a^			0.85 (0.61–1.17)
Race	White		reference
	Black		0.90 (0.13–6.09)
	Others		1.50 (0.41–5.48)
Education	<High school		reference
	High school		0.40 (0.11–1.52)
	Some college		0.59 (0.15–2.37)
	≥College		0.43 (0.11–1.72)
Working Status	Full-time working		reference
	Retired		0.48 (0.13–1.75)
	Homemaker		1.02 (0.29–3.59)
	Unemployment		0.87 (0.23–3.24)
Marital Status	Married		reference
	Separated		0.84 (0.09–8.13)
	Divorced		1.06 (0.38–2.93)
	Widowed		0.89 (0.23–3.43)
	Never married		1.43 (0.31–6.57)

* p<0.05.

** p<0.01.

a age was modeled as linear variables, with higher scores indicating higher age.

## Discussion

The results of this analysis showed that the effect of AB on the development of MD varied by type of AB. Financial support was harmful in terms of MD development, while other ABs (i.e., unpaid assistance and emotional support) were not associated with MD onset.

With regard to the harmful effect of financial support on MD development, previous research also showed that giving beyond one's own resources and feeling overwhelmed by others' demands had a stronger negative relationship with mental health [17]. The demand of financial support by others can be endless. Thus, financial support providers might be easily overwhelmed by constantly giving, and as a result, develop MD. Furthermore, the timing of receiving something in return for providing financial support may be delayed. That is, when people give some money to others, they expect something in return not necessarily from the receiver of the money, but some form of compensation for exhibiting good behaviors (i.e. financial support to others), such as reputation or status [18], [19]. On the contrary, people providing emotional support would receive rewards, such as sense of meaning or purpose, directly through providing emotional support, which reduces the likelihood of developing MD [20]. Thus, emotional support did not show significant negative effects on the development of MD, as the providing emotional support might be diluted by the rewards of contribution to improve others' emotion.

In addition, financial supporters might feel guilty, as it is suggested that the feeling of guilt promotes altruistic behavior [21]. Previous research also showed that the feeling of guilt, which is associated with religious strain, would lead to MD development [22]. That is, those who did not provide financial support don't feel guilt for not providing financial support, while those who provide financial support might feel guilt for not providing financial support [23]. As the amount of financial support is relative, those who feel guilt may not resolve their feeling of guilt through financial support. As the feeling of guilt is associated with negative emotions [24], financial support would have a harmful effect on the development of MD.

Previous research has shown the positive effect of providing financial support to children or grand children: for fathers or grandfathers who provide financial support were less likely to develop MD in later life [7]. The differential effect of providing financial support to others and family members can be explained by changes to the self-esteem. For fathers and grandfathers, they would think of themselves as fulfilling the father's obligation when providing financial support as they can provide the amount in which children or grandchildren need. On the contrary, it would be difficult to develop higher self-esteem through financial support to others, as the amount can be unlimited and is relative, as mentioned above [25]. However, the current study did not measure the feeling of guilt or self-esteem through AB among respondents. Further research looking at the psychological mechanism of the effect of financial support on MD is recommended.

In this research, 10 dollars was used as the cut-off of financial support for better distribution for analysis. Although the amount of 10 dollars or more a month is a fairly small amount, it was found that providing such a small amount had was associated with the development of MD. The distribution of the amounts given by the respondents among those who provide 10 dollar or more were: 10 dollar, 56.1%, 11–50 dollar, 33.3%, and 51 or more dollar (max: 100) were 10.6%. And, the percentage of developed MD in 2–3 years later were 21.6%, 18.2%, and 28.6%, respectively (Chi-square test: p = 0.84, data not shown). Thus, it is unlikely that outliers drive the findings.

On the contrary, although not statistically significant, unpaid assistance was in protective direction on MD onset. One possible explanation for the mental health benefits of unpaid assistance could be related to AB's nature of caring for others and focus outside of the self, which might nullify the self-focused nature of depression. Schwartz and Sendor proposed that the process of AB facilitates changing of internal standards, values, or conceptualization of quality of life [26]. However, previous studies showed limited evidence of the effect of volunteer activity on prevention of MD [16]. The differential effect on MD between unpaid assistance and volunteer activity might be due to the difference of focus, whether outside the self or not; people might join a volunteer activity from an achievement-oriented egocentricity, rather than focusing outside the self.

The current research has several limitations. First, the sample size was relatively small which might explain the marginal significance level of the effect of unpaid assistance and emotional support on MD. Further research using a larger representative sample is needed to replicate the findings. Second, AB used in this study was not validated in other studies. AB was measured numerically by type of AB (i.e. hours or dollars spent per month); however, whether the motivation of AB truly comes from an intention to do something for others was not measured. Third, the author could not adjust for all the possible covariates which influence the onset of MD. For example, if the author could include feeling of guilt or religious strain for doing AB as covariates, the results may change. Fourth, the author could not assess other types of AB, such as encouragement of others or giving useful information. As specific types of AB were associated with MD, it is needed to investigate the association between a wider range of AB and MD. Fifth, it is not known whether AB was done on demand or initiated by provider. If the AB was initiated by provider, the provider might feel a sense of control, which has a positive effect on mental health [27]. But if the AB was done on demand, the provider might not feel a sense of control. Sixth, with regard to outcome measures, it would be better to diagnose MD using DSM-IV, which is currently used. In addition, other types of mental disorders which are associated with psychological distress, such as generalized anxiety and panic disorders, were not used in the present study, as a follow-up to the MIDUS study measured MD only. Nonetheless, this study has several strengths which include being based on a nationally representative sample of middle aged adults in the US, its longitudinal design, the exclusion of those who have MD at baseline, and the use of a validated clinical diagnostic tool (CIDI-SF).

The findings of the effects of AB on MD have implications for mental health practice. First, randomized controlled trial to test the protective effect of unpaid assistance on development of MD needs to be implemented. In addition, the harmful effect of providing emotional and financial support should be considered as risk factors of MD in clinical and health care settings.

In conclusion, this study suggested the importance of AB on MD development. It is found that providing unpaid assistance had a protective effect on MD in 2–3 years later, while provision of emotional and financial support had harmful effect on MD. Further studies, including larger cohort studies or randomized controlled trials, are needed to elucidate the mechanism of the association between AB and MD.
